# Modeling Drug- and Chemical-Induced Hepatotoxicity with Systems Biology Approaches

**DOI:** 10.3389/fphys.2012.00462

**Published:** 2012-12-14

**Authors:** Sudin Bhattacharya, Lisl K.M. Shoda, Qiang Zhang, Courtney G. Woods, Brett A. Howell, Scott Q. Siler, Jeffrey L. Woodhead, Yuching Yang, Patrick McMullen, Paul B. Watkins, Melvin E. Andersen

**Affiliations:** ^1^Institute for Chemical Safety Sciences, The Hamner Institutes for Health SciencesResearch Triangle Park, NC, USA; ^2^Institute for Drug Safety Sciences, The Hamner Institutes for Health SciencesResearch Triangle Park, NC, USA

**Keywords:** systems toxicology, toxicity pathways, virtual liver, multi-scale modeling, drug toxicity, chemical toxicity, computational toxicology

## Abstract

We provide an overview of computational systems biology approaches as applied to the study of chemical- and drug-induced toxicity. The concept of “toxicity pathways” is described in the context of the 2007 US National Academies of Science report, “Toxicity testing in the 21st Century: A Vision and A Strategy.” Pathway mapping and modeling based on network biology concepts are a key component of the vision laid out in this report for a more biologically based analysis of dose-response behavior and the safety of chemicals and drugs. We focus on toxicity of the liver (hepatotoxicity) – a complex phenotypic response with contributions from a number of different cell types and biological processes. We describe three case studies of complementary multi-scale computational modeling approaches to understand perturbation of toxicity pathways in the human liver as a result of exposure to environmental contaminants and specific drugs. One approach involves development of a spatial, multicellular “virtual tissue” model of the liver lobule that combines molecular circuits in individual hepatocytes with cell–cell interactions and blood-mediated transport of toxicants through hepatic sinusoids, to enable quantitative, mechanistic prediction of hepatic dose-response for activation of the aryl hydrocarbon receptor toxicity pathway. Simultaneously, methods are being developing to extract quantitative maps of intracellular signaling and transcriptional regulatory networks perturbed by environmental contaminants, using a combination of gene expression and genome-wide protein-DNA interaction data. A predictive physiological model (DILIsym™) to understand drug-induced liver injury (DILI), the most common adverse event leading to termination of clinical development programs and regulatory actions on drugs, is also described. The model initially focuses on reactive metabolite-induced DILI in response to administration of acetaminophen, and spans multiple biological scales.

## Introduction

The 2007 report by the National Research Council (NRC) of the U.S. National Academies of Science, titled “Toxicity testing in the 21st Century: A Vision and A Strategy” (NAS/NRC, 2007), laid out a new path forward for the field of toxicology, envisioning an approach where most toxicity testing will be carried out *in vitro*, with a gradual reduction of reliance on high-dose animal studies. The basis of this risk assessment paradigm would be perturbation of cellular responses using a carefully selected suite of *in vitro* assays. Central to this vision is the idea of “toxicity pathways” – innate cellular signaling pathways that are perturbed by chemicals and pharmaceuticals, and the determination of chemical concentration ranges where those perturbations are likely to be excessive, thereby leading to adverse health effects if present for a prolonged duration in an organism. A key element of the proposed approach is the use of *computational systems biology models* as a tool to generate hypotheses about cellular level dose-response based on existing data sets, and to identify data and knowledge gaps that can help guide the design of *in vitro* assays, focused animal studies, and improved *in vitro – in vivo* extrapolation (IVIVE) methods.

In 2009, the U.S. Environmental Protection Agency published its Strategic Plan for Evaluating the Toxicity of Chemicals (U.S.EPA, 2009), which also envisions dynamic mathematical modeling as a key component of risk assessment linking toxicity pathways to dose-response. This plan calls for computational models that can predict organ injury from chemical exposure through simulation of: (i) the dynamic characteristics of exposure and dose; (ii) perturbations to molecular pathways; (iii) the link between these perturbations and alterations to cell state; and (iv) integration of molecular and cellular responses into a physiological “virtual tissue” (U.S.EPA, 2009).

Here we provide an introduction to some concepts relevant to developing computational systems biology models of intracellular toxicity pathways for environmental chemicals and pharmaceuticals, with specific relevance to toxicity of the liver. The peroxisome proliferator-activated receptor (PPAR)-α nuclear receptor (NR) pathway in primary human hepatocytes is used as an example for computational reconstruction of a toxicity pathway network from genomic data. We then use the example of aryl hydrocarbon receptor (AhR) activation in the liver to outline the process of developing a multi-scale spatial model of the liver lobule and interactions among multiple hepatic cell types consequent to exposure to toxic agents. Finally, we outline a predictive physiological model (DILIsym™) to understand drug-induced liver injury (DILI) in response to administration of acetaminophen, which spans multiple scales from the organ/tissue-level to the molecular and cellular levels. These varied modeling approaches, applied across different pathways and tissues, will be pivotal in creating twenty-first century *in vitro* toxicology testing strategies that are capable of determining likely pathway targets for chemicals and pharmaceuticals, and the risks associated with specific exposure and use conditions.

## Toxicity Pathways Underlying Biological Response to Chemicals

**Figure 1:**
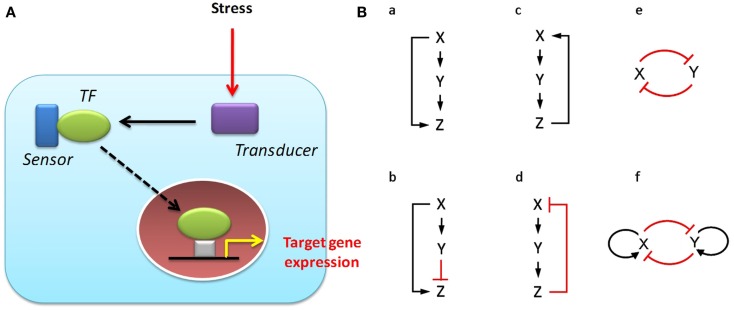
**Stress response pathways and network response motifs**. **(A)** Typical structure of a stress response pathway (adapted from Simmons et al., 2009). Canonical stress response pathways, conserved broadly across eukaryotes, have a common structure for sensing damage and launching a transcriptional response to counteract the stress. **(B)** Common network motifs in intracellular response pathways. Three elements (genes/proteins) X, Y, and Z, in a pathway can regulate each other to form: (a) a coherent feed-forward loop where X activates Y, and both X and Y activate Z; (b) an incoherent feed-forward loop where X activates both Y and Z, but Y suppresses Z; (c) a positive feedback loop; and (d) a negative feedback loop. Two transcription factors X and Y can regulate each other through, for instance: (e) a double-negative feedback loop; or (f) a double-negative feedback loop with positive autoregulation. Sharp arrows denote activation; flat arrows denote suppression.

The biological effects of a drug or hazardous chemical (ligand) in individual cells are mediated by cell-membrane or cytosolic “receptor” molecules and downstream signaling and transcriptional networks, which together comprise intracellular *toxicity pathways*. Changes in the topology and dynamic behavior of these pathways subsequent to recognition of the external ligand account for the particular shape of the dose-response curve for specific phenotypic end points. A finite number of core “stress response pathways” mediate the response of cells to various chemical stimuli to maintain homeostasis, or to make specific cell-fate decisions such as proliferation, differentiation, or apoptosis (Simmons et al., 2009). Examples of stress response pathways include the oxidative stress response, heat-shock response, DNA-damage response, hypoxia, and endoplasmic reticulum stress pathways, all of which are present in all cell types of an organism, and feature a common architecture consisting of a transcription factor (TF), a “sensor,” and a “transducer” (Figure 1A; Simmons et al., 2009). This suite of pathways is typically activated at concentrations of chemicals significantly lower than those that lead to adverse effects at the organism level, and can be assayed as a group to serve as predictors of potential cell damage (Kultz, 2005; Simmons et al., 2009). A second group of toxicity pathways is comprised of the signaling networks related to activation of specific endogenous receptor pathways, such as estrogen, androgen, and thyroid hormone signaling. Over-stimulation or inhibition of these diverse pathways can lead to toxic outcomes.

The canonical toxicity pathways discussed above are in turn made up of a core set of functional *regulatory network motifs* that underlie cellular homeostasis and fate decisions including phenotypic transitions (Alon, 2007). Each of these regulatory motifs, originally discovered from detailed investigation of transcriptional regulatory networks in the bacterium *Escherichia coli* (Shen-Orr et al., 2002) and the budding yeast *Saccharomyces cerevisiae* (Lee et al., 2002), has a characteristic structure and the capacity to perform specific information-processing functions (Bhalla and Iyengar, 1999; Tyson et al., 2003; Alon, 2007; Figure 1B). Some examples of response motifs are:

(i) *negative feedback*, which enables homeostasis and acceleration of response time in gene circuits (Rosenfeld et al., 2002; Zhang and Andersen, 2007);
(ii) *positive feedback*, which generates switching behavior between multiple phenotypic states (Ferrell, 2002);
(iii) the *coherent feed-forward loop*, which can introduce a time delay in activation as well as detect persistence in the activating signal (Mangan et al., 2003); and
(iv) the *incoherent feed-forward loop*, which can function as a pulse generator and response accelerator (Mangan et al., 2003, 2006).

These motifs often occur in combination to generate more complex regulatory patterns in transcriptional networks (Alon, 2007). Response motifs have been identified not just in unicellular organisms but also in the cells of higher organisms – for example in the circuits that control gene expression in the pancreas and the liver (Odom et al., 2004) as well as the regulatory circuits of human embryonic (Boyer et al., 2005) and hematopoietic (Swiers et al., 2006; Rothenberg, 2007) stem cells. Perturbation of these regulatory motifs is likely to be a key element of toxic response, and a better understanding of their organization and dynamic behavior should lead to improved prediction of the cellular outcome of specific perturbations introduced by various chemicals.

## Computational Systems Biology Models to Understand Perturbations in Toxicity Pathways

Detailed characterization of molecular signatures associated with cellular perturbation of key toxicity pathways and disease states has been made possible by the advent of the “-*omics*” era. However, these molecular signatures do not by themselves translate to a clear causal network of pathway perturbation. Rigorous quantitative analysis of specific pathways and network motifs derived from these large-scale molecular signatures will aid mechanistic understanding of the underlying biological processes (Araujo et al., 2007). In particular, understanding the dynamic, dose-dependent behavior of toxicity pathways will require stimulation of these pathways at a number of time points and at various concentrations of the activating chemical, rather than static snapshots of the molecular state (Danna and Nolan, 2006).

*Computational systems biology pathway (CSBP) models* can play a key role in this process, allowing mechanistic prediction of the dose-response based on pathway dynamics (Zhang et al., 2010a). These models will have to be based on molecular circuits responsible for the basal operation of normal cellular pathways in the absence of an external chemical stressor, which sets up the background state from which additional perturbations will occur as the stressor level increases. A properly implemented CSBP model would take such changes into account to predict the range of concentrations of stressors that would not produce appreciable adversity. A key aspect of the applicability of such models is the quantitative characterization of the underlying molecular circuits from appropriately designed *in vitro* assays. CSBP models can also allow the assessment of pathway components that display polymorphisms in the human population to help identify sensitive subpopulations.

*Deterministic simulations* based on ordinary differential equations (ODEs) are a common approach to modeling dynamical systems like intracellular signaling circuits. An assumption with deterministic ODE models is that the molecular components of the network of interest exist in a well-mixed volume such as the cytosol or nucleus, and that the amounts or concentrations of all molecular species in the network can be approximated by continuous variables. A typical deterministic model consists of a set of coupled ODEs, each describing the rate of change in the concentration or abundance of a molecular component, and incorporating terms that account for the known biochemical interactions among the various molecular species (Aldridge et al., 2006). The numerical values of parameters and initial conditions are assigned based on existing literature and *in vitro* data, and the time course of the system is simulated using one of a variety of numerical ODE solvers. Parameter assignment is not always a straightforward exercise: experimental data is often not available for the particular species or cell type being modeled. In such cases, parameter estimation techniques need to be applied (Swameye et al., 2003). Parameter uncertainty, distinct from biological variability, can cause uncertainties in prediction from a computational model (Vanlier et al., 2012). Although this ODE-based approach does not take into account either spatial diffusion or noise in gene expression, it is a valuable computational tool that has provided many insights into the design and function of molecular circuits underlying a number of biological processes like cell cycle regulation, signal transduction, cell differentiation, stress response, and biological rhythms (Carrier et al., 1995; Bhalla et al., 2002; Forger and Peskin, 2003; Novak and Tyson, 2003; El-Samad and Khammash, 2006; Bhattacharya et al., 2010).

Stochastic fluctuations in gene expression and levels of intracellular molecular species, which are ignored in the ODE-based deterministic modeling approach, can play an important role in cellular response by generating non-genetic phenotypic variability among an isogenic cell population (Kaern et al., 2005; Losick and Desplan, 2008; Pearson, 2008). The random fluctuations in mRNA and protein concentrations can be modeled by *stochastic simulation* algorithms like Gillespie’s direct method and first-reaction method (Gillespie, 1976, 1977). Gillespie’s algorithm is essentially a Monte Carlo simulation technique where the number of reacting molecules in the model system and the reaction rate constants are used to generate two probability density functions, one of which predicts the time interval between successive reaction events, and the other identifies which one among all possible reactions is likely to occur next. The time variable in the simulation is then updated by the calculated time interval, and the copy numbers of the reactants and products of the predicted reaction are updated according to the reaction stoichiometry. Several modified versions of the original Gillespie algorithm have been developed to improve its computational efficiency, including the next reaction method, tau-leaping, and hybrid models (Gibson and Bruck, 2000; Gillespie, 2000; Rathinam et al., 2003; Salis and Kaznessis, 2005). Most of these are approximate methods that greatly reduce the simulation time. In some cases, stochastic simulations simply add “white noise” terms to the most variable species in ODE equations. Applications of stochastic modeling tools include investigation of oscillatory patterns in protein levels (Proctor and Gray, 2008) and cellular differentiation (Zhang et al., 2010b).

Developmental processes are usually driven by discrete, all-or-none changes in the expression of lineage-specific genes belonging to a large gene regulatory network. The *Boolean network modeling* paradigm, where each variable (gene) is assumed to take either of two values, 0 (off) and 1 (on), is often a good approximation of the gene expression patterns in these processes. The state of each gene is updated according to its current state and that of other regulatory genes it is connected to in the network, as governed by a preset Boolean logical rule table. This binary-state assumption reduces the dependence on various kinetic parameters in the model, instead making full use of the large available database of qualitative protein–protein and protein–gene interactions. As with deterministic and stochastic models, a simulation of a Boolean network model should converge to an attractor state representing the binary gene expression pattern of a particular phenotypic state (Albert and Othmer, 2003). For chemicals that exhibit developmental toxicity, a Boolean network model can be used to predict low-dose effects based on high-throughput screening, allowing comparison of gene expression profiles between the undisturbed and disrupted states of the transcriptional regulatory network (Jack et al., 2011).

These various modeling techniques are based on a topological representation of cellular signaling networks, and ignore the spatial relationship among intracellular molecular species and the spatial heterogeneity inside a cell. In reality, the cytosol, the nucleus and organelles such as mitochondria and endoplasmic reticulum segregate the intracellular space into a number of discrete compartments. The difference in concentrations of molecules between these compartments and inter-compartment molecular traffic can be accounted for by simple compartmental models, where each subcellular compartment is assumed to behave as a well-mixed sub-system. However, diffusion must be explicitly considered in cases where the spatial aspect of molecular diffusion within cellular compartments becomes rate-limiting. Examples include pattern formation in the animal body in response to concentration gradient of morphogens, effects of chemicals on different dermal layers when absorbed by the skin, and propagating waves of signaling molecules in the cytosol. In these circumstances, *spatiotemporal models* based on partial differential equations may be employed (Kholodenko, 2006; Kholodenko et al., 2010). The spatial dimension can also be explicitly incorporated by modeling the motion and interaction of distinct molecular species as discrete particles, for example with agent-based spatial modeling approaches. Agent-based models also address another problem arising from the lack of a spatial component in network models: incorporating multicellular or tissue-level interactions.

### The agent-based modeling approach

**Figure 2:**
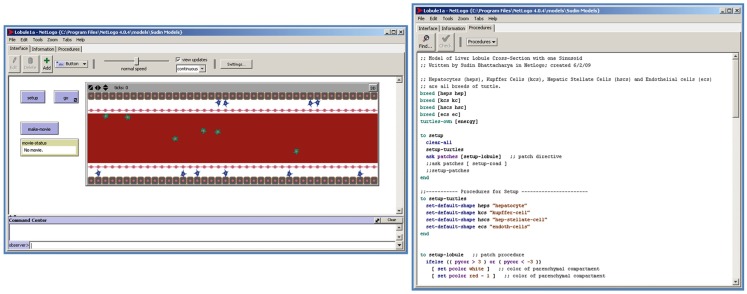
**The NetLogo agent-based modeling platform with an integrated visualization/programing interface**. “Agents” occupy a virtual spatial “world” in this two-dimensional representation of the liver lobule (left). The programing interface (right) in NetLogo makes it simple to test various changes in model code during model development.

*Agent-based modeling* (ABM), also referred to as “individual-based modeling” (Bonabeau, 2002; Grimm et al., 2005; An et al., 2009) is a more intuitive approach than traditional equation-based modeling formalisms, and as such can be helpful in model development, model interpretation, and model use by a variety of stakeholders. The “agents” in an ABM may be individual molecules, cells, or other entities that populate a virtual “world” (a discrete lattice), with each agent represented as a distinct data structure (or “object”) in the computational model (see Figure 2). Agents can move in the physical space of the world, and interact with neighboring agents according to a pre-defined set of rules. As the model is simulated over a large number of iterations, these local interactions generate macroscopic, sometimes counterintuitive, phenomena of interest – referred to as “emergent properties” of the system being modeled.

Agents in an ABM are modeled as discrete entities located in a physical space – thus it is no longer necessary to assume a well-mixed continuous system as in differential equation-based dynamic modeling approaches. The reliance on averaged aggregate parameters is therefore reduced in favor of an emphasis on strict definition of rules governing agent behavior and interactions. Decisions regarding such explicit rules for agent behavior are typically more intuitive than the choice and estimation of abstract parameters in equation-based models, making it easier for decision-makers to interpret and use the model. ABM platforms like NetLogo (http://ccl.northwestern.edu/netlogo/) use an integrated visualization/programing interface (Figure 2), which makes it straightforward to evaluate the effect of modifications to the “engine” of the model on model behavior.

Agent-based modelings were first used in models in social science and ecology, but have been applied to a wide range of biological problems in recent years, particularly for modeling pathophysiological processes with a significant spatial component (An et al., 2009). These include tumor formation (Engelberg et al., 2008; Gerlee and Anderson, 2009; Zhang et al., 2009b,c), inflammation (An, 2001, 2008), wound healing (Walker et al., 2004; Vodovotz, 2006; Sun et al., 2007), T-cell activation and proliferation within a lymph node (Bogle and Dunbar, 2010), and stromal cell trafficking during acute skeletal muscle ischemia (Bailey et al., 2009). Agents in these “virtual tissue” models represent the behavior of individual cells – the natural functional unit in tissue-level biological phenomena. In the toxicology context, virtual tissues can be thought of as multicellular models of tissue microenvironment that attempt to reconstruct the *in vivo* milieu of target organs to simulate the physiological consequences of toxicity pathway activation by specific chemicals. As such they represent an extension of traditional compartmental models to the scale of individual cells in a tissue (Shah and Wambaugh, 2010).

## Liver Modeling Case Studies

### Case study 1: A CSBP model: Causal transcriptional network inference

The 2007 NAS report (NAS/NRC, 2007) emphasized computational modeling of core intracellular toxicity pathways as a crucial component of the new toxicity testing paradigm. While these pathways have been the object of a large number of experimental studies, the causal molecular networks giving rise to activation of the pathways have not been mapped out in sufficient detail. Here we describe an approach for causal network mapping we are currently applying to the analysis of two prototype toxicity pathways: the PPARα pathway in liver parenchymal cells, and the estrogen receptor pathway in uterine epithelial cells. Such approaches are likely to be pertinent to mapping the pathways activated by a broad group of toxicants.

Both ER and PPARα belongs to the NR family: ligand-activated TFs that regulate a variety of physiological functions involved in development, metabolism, and homeostasis. These include the steroid hormone-receptors: estrogen receptor, androgen receptor (AR), glucocorticoid receptor (GR), and progesterone receptor (PR), as well as the PPARs, liver X receptors (LXRs), retinoic acid receptors (RARs), and retinoid X receptors (RXRs). NRs act on chromatin combinatorially and dynamically throughout the genome to regulate transcriptional responses to physiological and environmental stimuli (Carlberg and Seuter, 2010; George et al., 2011).

The PPARs function as regulators of hepatic lipid metabolism and adipogenesis (Kersten et al., 2010; Pyper et al., 2010; Siersbæk et al., 2010). They form heterodimers with RXR and bind to peroxisome proliferator response elements (PPREs) on the promoters of target genes to induce gene expression. There are three identified PPAR isotypes α, β, and γ – among which PPARα regulates genes involved in fatty acid oxidation, ketogenesis, gluconeogenesis, cholesterol catabolism, and lipoprotein metabolism (Mandard et al., 2004; Lefebvre et al., 2006), as well as anti-inflammatory response (Zandbergen and Plutzky, 2007; Michalik and Wahli, 2008). Sustained PPARα-mediated induction of peroxisome proliferation can produce liver tumors in rats and mice (Reddy et al., 1976, 1980).

In the canonical picture, activation of PPARα in liver parenchymal cells causes downstream alterations in gene expression through a series of coordinated steps:

(i) phosphorylation of PPARα in the cytosol;
(ii) translocation of PPARα to the nucleus;
(iii) heterodimerization with its binding partner retinoid X receptor alpha (RXRα);
(iv) binding of the heterodimer at DNA-response-elements (PPREs) in the promoters of target genes; and
(v) alterations in binding of co-activators and co-repressors.

In recent years, genome-wide profiling of TF-binding activity has provided an unprecedented global picture of gene regulation. Chromatin immunoprecipitation (ChIP) in combination with microarray hybridization (ChIP-chip) or high-throughput sequencing (ChIP-seq) has been widely used for genome-wide location analysis and characterization of TF-chromatin interactions both for NRs and other TFs (Odom et al., 2004; Carroll et al., 2005; Bieda et al., 2006; Gao et al., 2008; John et al., 2008; Lefterova et al., 2008; Nielsen et al., 2008; Fullwood et al., 2009; Delacroix et al., 2010; Hu et al., 2010; Ravasi et al., 2010; van der Meer et al., 2010; Dere et al., 2011). An important finding of these studies is the combinatorial control of gene expression by NRs. TF-binding sites are often clustered in the genome, which allows coordinated action of multiple TFs to induce or suppress the expression of individual genes in a cell type and condition-specific manner (George et al., 2011).

In addition, gene regulation by “DNA-independent” chromatin-NR interactions is surprisingly common across the genome (George et al., 2011), whereby NRs indirectly modulate transcription by “tethering” to other TFs directly bound to DNA. About 25% of ER and 30% of GR binding to the chromatin, for example, appears to be DNA-independent – possibly enabled by tethering to co-localized TFs like RUNX1 and AP1 (Heldring et al., 2007; So et al., 2007; Reddy et al., 2009; Stender et al., 2010). Although the number of potential NR binding sites across the genome is vast, only a small fraction of these sites is occupied, with an even smaller number of sites likely contributing to functional gene regulation *in vivo* (Bourdeau et al., 2004; Carroll et al., 2005, 2006). These observations suggest that a more realistic picture of NR-mediated gene regulation can be obtained by combining gene expression data from transcriptome profiling with genome-wide analysis of NR localization.

Accordingly, we are using a combination of: (i) microarray-based gene expression data; (ii) published ChIP-on-chip analyses of genome-wide NR binding; and (iii) curated lists of directly bound NR target genes to develop a comprehensive picture of NR-mediated transcriptional regulation. Differentially expressed genes responding to specific NR ligands are classified into three groups:

(i) genes with the NR directly bound to their promoters;
(ii) genes with the NR indirectly bound to their promoters by tethering to other directly bound TFs
(iii) non-NR-bound genes regulated by other TFs.

**Figure 3:**
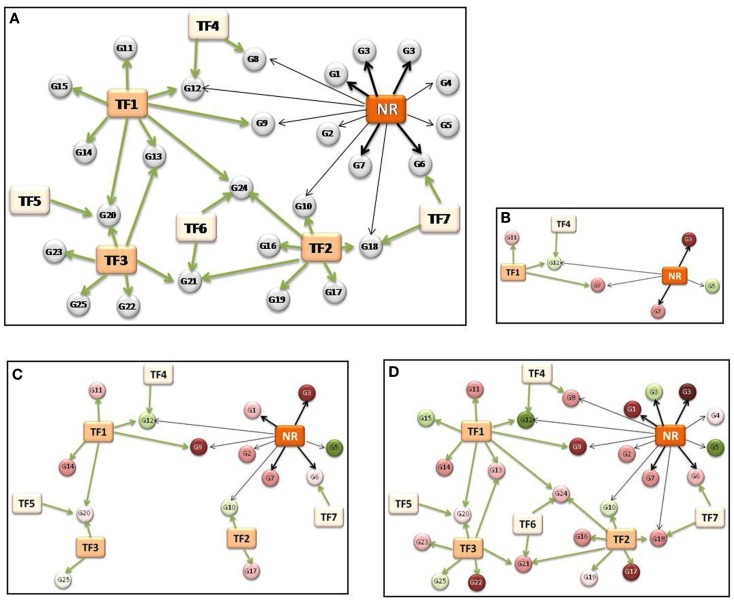
**Schematic representation of inferred nuclear receptor transcriptional regulatory network and dose-response**. Rectangular nodes indicate regulatory transcription factors (TFs), with the functional nuclear receptor (NR) marked in dark orange. Each directed edge in the network indicates binding of a TF to the promoter of a target gene (circular nodes, G1–G25). Dark black arrows connect NR with direct targets; light black arrows with indirect targets. Green arrows connect other (non-NR) TFs with their target genes. **(A)** The “latent” network, showing well-connected transcriptional “hubs” (NR, TF1, TF2, TF3). **(B–D)** Evolution of network structure with increasing levels of stimulation with NR ligand. Target genes are colored by level of expression (red: upregulation; green: downregulation).

The non-NR TFs that regulate genes in groups (ii) and (iii) are identified from the TRANSFAC (Biobase Corporation, Beverly, MA, USA) database of TF-DNA interactions. These transcriptional interactions may be summarized in a “latent regulatory network” (shown schematically in Figure 3A), which could potentially reveal the significant regulatory hubs in the transcriptional network. Superposition of gene expression results from microarray studies onto the latent network then allows visualization of time- or dose-dependent transitions in the network (Figures 3B–D). This derivation focuses purely on regulation at the transcriptional level, and as such ignores epigenetic-level regulations including post-translational modifications of NRs that could be important in certain dosing contexts.

### Case study 2: Agent-based model of the human liver

#### Motivation for multi-scale, agent-based models of the liver

Multi-scale spatial models based on the ABM formalism are particularly suited for investigating the effects of toxic chemicals and drugs in the liver, which arise from a combination of cellular and tissue-level mechanisms, with marked heterogeneities observed across the liver lobule. These mechanisms are discussed below in the context of dioxin-induced liver toxicity – a particularly well-studied phenomenon.

**Figure 4:**
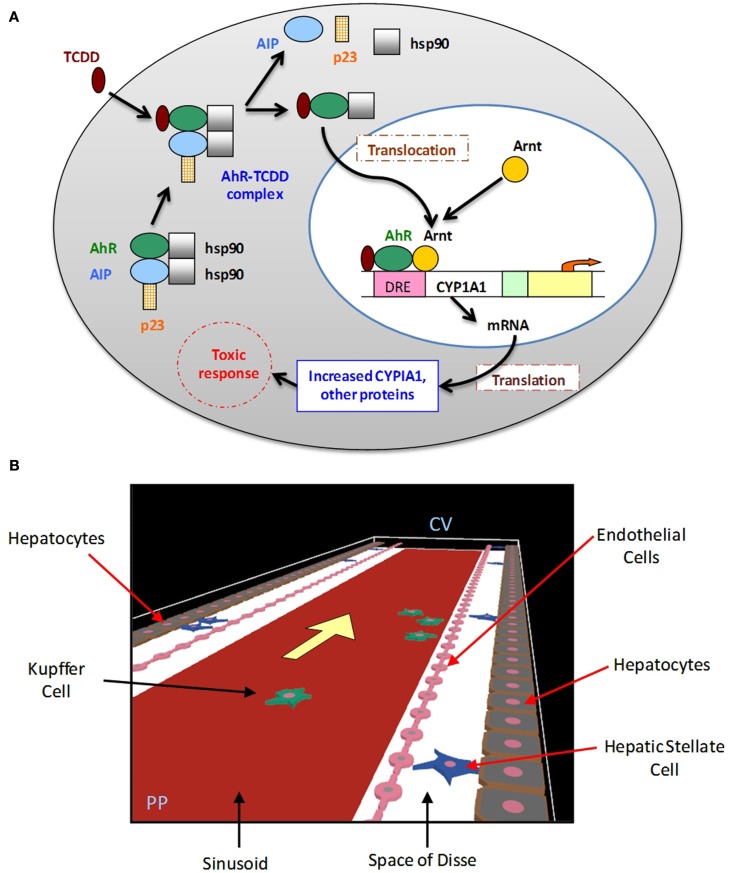
**The AhR signaling pathway, and agent-based spatial model of liver lobule**. **(A)** Key components and events in AhR signaling pathway activation. *AIP:* Aryl hydrocarbon receptor interacting protein; *hsp90:* heat-shock protein 90; *Arnt:* aryl hydrocarbon receptor nuclear translocator; *DRE:* dioxin-responsive element. **(B)** Agent-based model of liver lobule section (representational unit: one sinusoid), incorporating hepatocytes, liver endothelial cells, hepatic stellate cells, and Kupffer cells.

The persistent environmental contaminant 2,3,7,8-tetrachloro dibenzo-*p*-dioxin (TCDD) belongs to a class of toxicants known as halogenated aromatic hydrocarbons (HAHs). The toxic effects of HAHs in mammals are mediated through binding to the AhR (Poland and Knutson, 1982; Schmidt and Bradfield, 1996; Rowlands and Gustafsson, 1997). The liver is one of the most sensitive organs for toxicity induced by TCDD. A spatial agent-based “virtual tissue” model of the liver lobule that incorporates a mechanistic representation of the activation of the AhR toxicity pathway in individual hepatocytes can be used to investigate the sequence of events leading from early activation of the AhR pathway through subsequent cellular effects to cell proliferation, and culminating in liver cancer as an endpoint. The structure of the AhR pathway is well-studied (Figure 4A), and as such could serve as a good case study for multi-scale, quantitative dose-response model development based on information from *in vitro* assays and *in vivo* biomarkers.

The hepatic dose-response of TCDD culminating in liver cancer consists of the following key steps (Mills and Andersen, 1993):

(i) Accumulation of TCDD in the target tissue.
(ii) Formation of a complex with AhR.
(iii) Activation of growth-regulatory genes by the AhR-TCDD complex.
(iv) Cellular responses to the altered expression of growth-regulatory gene products.
(v) The effect of these cellular events on tumor promotion and progression.

However the mechanistic and causal links connecting steps (iii) through (v) are not well understood.

The carcinogenic effects of TCDD are believed to be mediated by tumor promotion rather than initiation (Pitot et al., 1980). A “negative selection” model of tumor promotion has been proposed where specifically mutated cells acquire a proliferative advantage in the presence of persistent mitosuppression (Andersen et al., 1995). TCDD suppresses apoptosis induced in rat hepatocytes by DNA-damaging agents, which could result in selective expansion of clones evading growth arrest and apoptosis (Worner and Schrenk, 1996; Bock and Kohle, 2005). The traditional benchmark dose calculation for low-dose hepatotoxic effects of TCDD is linked to centrilobular induction of cytochromes P450 1A1 (CYP1A1) and 1A2 (CYP1A2). However the relation between these early centrilobular gene expression events, and subsequent cell proliferation events likely originating in the periportal region of the liver lobule, is unclear. Specifically, cytochrome P450 activity and cell proliferation follow different dose-response patterns: CYP1A1 activity appears to be reversible following prolonged TCDD exposure, while the selective growth of altered hepatic foci and cell proliferation are persistent (Maronpot et al., 1993; Sewall et al., 1995; Tritscher et al., 1995; Whysner and Williams, 1996; Viluksela et al., 2000). Liver cytotoxicity may be an intermediate step in the sequence of cellular events leading to tumor promotion (Busser and Lutz, 1987; Maronpot et al., 1993; Whysner and Williams, 1996; Viluksela et al., 2000).

Putative liver stem cells – known as “oval cells” in the rat and “progenitor cells” in the human, may be one possible link between early events in AhR toxicity pathway activation and eventual cell proliferation culminating in liver cancer (Lemire et al., 1991; Libbrecht et al., 2001). These cells, which can differentiate into either hepatocytes or cholangiocytes (bile duct cells; Roskams et al., 2003), may have a role in development of human liver tumors (Libbrecht et al., 2001). In a quiescent or healthy liver, oval cells are localized in the Canals of Hering, situated in the smallest branches of the biliary tree close to the periportal end of the liver lobule (Fausto and Campbell, 2003; Fausto et al., 2006; Gaudio et al., 2009). However in the diseased human liver, in the case of both hepatitis (Libbrecht et al., 2000) and hepatocellular adenomas (Libbrecht et al., 2001), progenitor cells and hepatocyte-like cells are found scattered throughout the parenchyma, suggesting migration and differentiation toward the hepatocyte lineage. Intriguingly, biopsies of human primary liver tumors have revealed cells with an intermediate phenotype between that of hepatocytes and bile duct cells, suggesting the involvement of these liver progenitor cells (Robrechts et al., 1998; Kim et al., 2004).

The Hippo signaling pathway regulates cell contact inhibition and suppression of hepatic oval cell proliferation (Zeng and Hong, 2008; Lee et al., 2010). Interestingly, TCDD activates the proto-oncogene cyclin A to deregulate contact inhibition in rat liver oval cells (Weiss et al., 2008), providing a possible role for this pathway in TCDD-induced tumor promotion. Several other non-parenchymal cells (e.g., hepatic stellate cells and Kupffer cells) also regulate oval cell activity (Zhang et al., 2009a). Livers of rats treated with TCDD and other AhR agonists exhibit loss of cell-cell contact and enhanced cell proliferation including oval cell hyperplasia (Chramostová et al., 2004; NTP, 2006a,b; Andrysík et al., 2007; Dietrich and Kaina, 2010).

In spite of the large number of empirical studies with TCDD, some of which are summarized above, there is no agreement on a unifying hypothesis to connect these observations into a mechanistic description linking AhR toxicity pathway activation to liver cancer. Spatial multicellular ABM of the liver lobule incorporating parenchymal (hepatocytes) and non-parenchymal cells (hepatic stellate cells and Kupffer cells), along with oval cell proliferation, can serve as an ontological tool to assemble diverse *in vitro* and *in vivo* observations and compare alternative hypotheses for TCDD-induced tumor promotion. Research teams at The Hamner are pursuing multi-scale modeling approaches for examining pathways perturbed by TCDD and other environmental chemicals, as well as therapeutic molecules that cause DILI.

#### The liver ABM

A realistic spatial model of the liver lobule and drug/chemical-induced toxic effects needs to account for:

(i) cellular heterogeneity across the lobule;
(ii) multiple cell types in the liver lobule that participate in liver injury.

**Figure 5:**
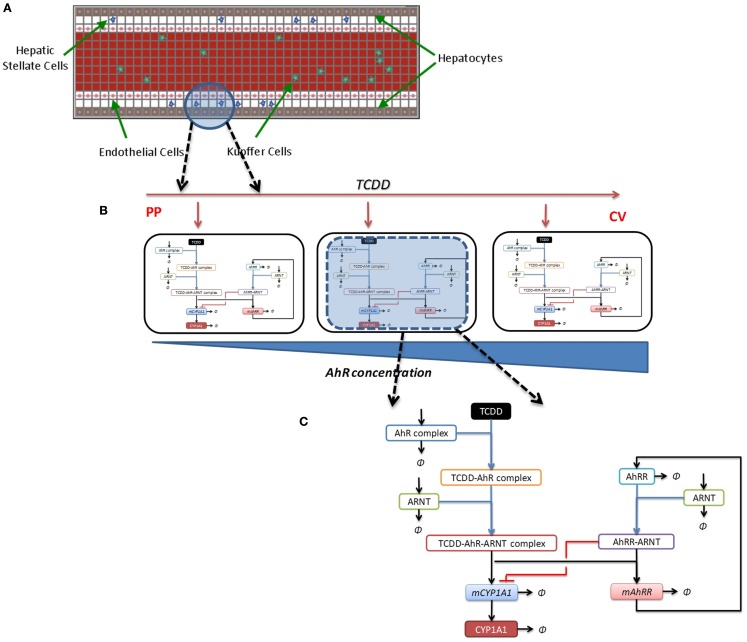
**Implementation of multi-scale agent-based model (ABM) of liver lobule**. **(A)** ABM of the lobule showing distinct cell types, with kinetics from PBPK model used as input for TCDD flow rate through the lobule. **(B)** Individual agents (hepatocytes) along the lobule, with heterogeneous states (e.g., with graded AhR concentration), will be exposed to TCDD molecules flowing through lobule sinusoid, which determines the dose for each agent (PP: periportal end; CV: central-vein end). **(C)** Each agent (hepatocyte) is implemented as a molecular network: in this case the intra-hepatocyte cascade of events linking ligand (TCDD) – activation of the Ah receptor to expression of cytochrome (CYP) P450 proteins. [**(C)** adapted from Gim et al., 2010].

The virtual tissue formalism can be used to develop a spatial agent-based model of the human liver lobule (the “lobule ABM”), with individual hepatocytes represented by single agents. There have been some initial efforts toward development of agent-based representations of the liver (Hunt et al., 2006; Davila and An, 2010; Wambaugh and Shah, 2010). Here we lay out the steps toward developing a multi-scale model, where the lobule ABM is coupled with an intra-hepatocyte ODE-based kinetic model of AhR pathway activation for dose-response prediction with TCDD or other chemicals (Figures 5A–C). A preliminary version of such a model, incorporating hepatocytes, liver endothelial cells, hepatic stellate cells, and Kupffer cells, is shown in Figure 4B. Other components, including oval cells, will be added in course of model refinement.

(i) Output from a standard TCDD physiologically based pharmacokinetic (PBPK) model (e.g., Leung et al., 1988, 1990) estimates disposition of TCDD in the liver lobule: the input dose for the lobule ABM (Figure 5A).
(ii) This TCDD input dose acts upon the individual agents (hepatocytes) in the model, which occupy heterogeneous states by virtue of differential gene expression: e.g., graded expression of the Ah receptor along the lobule (higher at the centrilobular end; Lindros et al., 1997; Figure 5B).
(iii) The agents (hepatocytes) are implemented as intracellular signaling cascades: i.e., key molecular events associated with the AhR toxicity pathway (Figures 4A and 5C). Individual signaling events along the cascade can be modeled and quantitatively calibrated from the literature, including TCDD-AhR binding (Poland and Knutson, 1982), TCDD-AhR-ARNT binding (Rowlands et al., 1996; Rowlands and Gustafsson, 1997), activation of AhRR (AhR repressor) by liganded AhR and reciprocal inhibition of AhR binding activity by AhRR (Mimura et al., 1999; Evans et al., 2008), and cytochrome (CYP) P450 protein induction (Jones et al., 1985; Fisher et al., 1989; Nebert et al., 2004).

Subsequent steps incorporate additional events into the signaling cascade shown in Figure 5C, for example crosstalk between the AhR and cell cycle/cell proliferation pathways (Elferink, 2003; Dietrich and Kaina, 2010). The tissue-level model implements specific “agent rules,” e.g., hepatocyte proliferation (agent addition), or hepatocyte death (agent deletion), at specific levels of TCDD, or downstream signaling components/metabolites. The model structure would be extensible to other chemicals besides TCDD; however the implementation of the agent structure (i.e., the signaling cascade shown in Figure 5C) will vary from case to case, depending on the specific toxicity pathways stimulated. In addition, a variety of interactions between diverse cell types/agents will be specified to account for pharmacodynamic responses, including necrosis, apoptosis, proliferation, and tumor promotion.

### Case study 3

#### ODE-based mechanistic multi-species model of the liver

**Figure 6:**
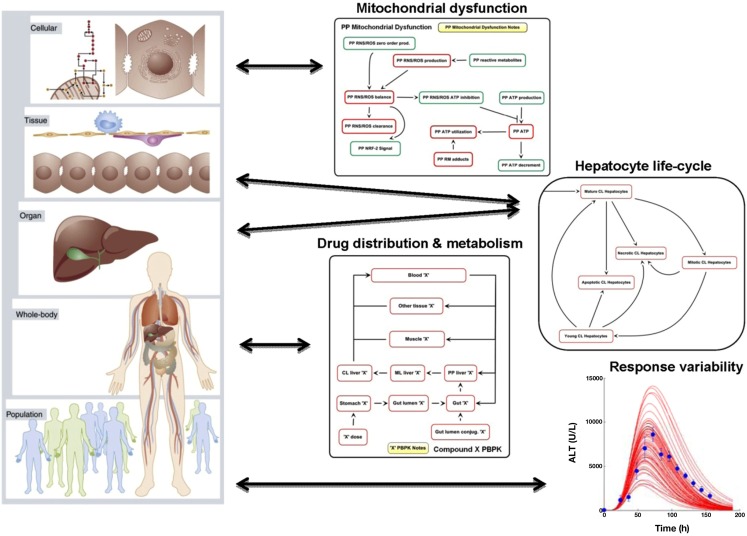
**Conception of the multi-scale DILIsym™ model**. The DILIsym™ model is a multi-scale representation of liver physiology, encompassing molecular and cellular interactions, variability in different zones of the liver acinus, whole-body drug distribution and metabolism, as well as variability in both drug profile and underlying physiology leading to alternate responses. The multi-scale graphic has been reprinted with permission from Kuepfer (2010).

*Drug-induced liver injury* is the most common adverse drug event leading to termination of clinical development programs and regulatory actions on drugs, as well as the most common cause of acute liver failure in the United States (Ostapowicz et al., 2002). The DILI-sim Initiative is a partnership of several drug development companies, led by The Hamner Institutes for Health Sciences, to improve prediction and understanding of DILI. The DILI-sim Initiative sponsors the development and application of the DILIsym™ model, a computational representation of physiological processes involved in DILI. The model (Howell et al., 2012; Woodhead et al., 2012) initially focuses on reactive metabolite-induced DILI and spans multiple scales of physiology, from the organ/tissue-level to the molecular and cellular levels (Figure 6). The DILIsym™ model utilizes ODEs in the MATLAB computing platform (The MathWorks, Natick, MA, USA). Multiple sub-models are included: (a) PBPK dynamics, (b) glutathione (GSH) depletion and synthesis, (c) mitochondrial dysfunction, (d) the hepatocyte life cycle and cell death due to ATP depletion and mitochondrial dysfunction, (e) the innate immune response, and (f) clinical endpoints, e.g., bilirubin, alanine aminotransferase (ALT), and keratin 18. Using publicly available literature, the DILIsym™ model includes parameters to represent mouse, rat, and human physiology which enables species-specific investigation and facilitates cross-species interpretation. Further, a genetic algorithm has been applied to create alternate parameterizations of the model within each species. These alternate parameterizations, termed SimPops™, are generated to explore inter-individual differences in response with respect to DILI. Simulated protocols run in the SimPops™ framework allow the researcher to assess whether and how variation in the underlying biology impacts the predicted outcomes. The model integrates available mechanistic data on DILI to recapitulate *in vivo* responses using only *in vitro* data, and to identify critical drug-related uncertainties that if resolved could vastly improve the understanding and/or treatment of drug hepatotoxicity. The following examples illustrate the application of the DILIsym™ model to understand DILI induced by acetaminophen (APAP) and methapyrilene (MP).

**Figure 7:**
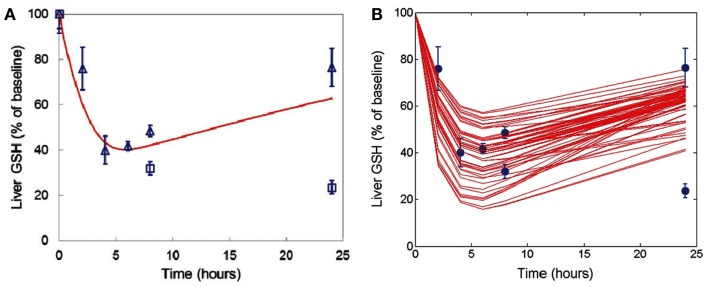
**Simulations from the DILIsym™ model are compared against published data on the underlying biology as illustrated in the GSH example**. **(A)** The baseline simulated rat administered 500 mg/kg APAP was evaluated for degree and kinetics of GSH depletion against data from Chen et al. (2009) (squares) and Vendemiale et al. (1996) (triangles). **(B)** A genetic algorithm was applied to created alternate simulated rats with variability in multiple parameters. Results from alternate simulated rats administered 500 mg/kg APAP were compared against the same data (circles for all data points). Alternate simulated rats reflect reported biological variability and permit testing of how such biological variability impacts outcomes.

**Figure 8:**
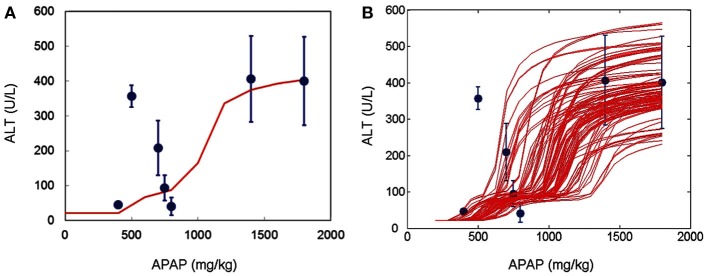
**Simulations from the DILIsym™ model are compared against published data on indicators of liver damage in response to APAP**. **(A)** Results from the baseline simulated rat administered different doses of APAP was evaluated for ALT elevation against data from multiple references (Zieve et al., 1985; Chanda et al., 1995; Sugimura and Yamamoto, 1998; Wang et al., 1999; Waters et al., 2001; Gueguen et al., 2007; Chen et al., 2009; all data in circles). **(B)** Results from alternate simulated rats administered different doses of APAP were compared against the same datasets. Alternate simulated rats reflect reported variability in liver damage following APAP administration.

**Figure 9:**
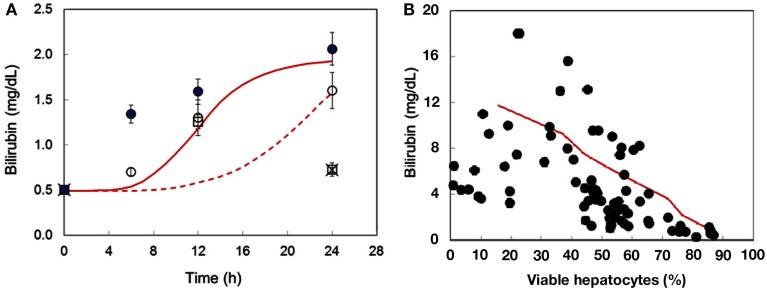
**Alternate hypotheses for mechanisms underlying early drug-induced elevation in bilirubin were tested using the DILIsym™ model**. **(A)** Drug was simulated in the presence (solid line) or absence (dashed line) of an ATP effect on bilirubin formation. Simulation results were compared with published data (Pooranaperundevi et al., 2010a,b), closed and open circles (Nirala and Bhadauria, 2008), x mark (Sawant et al., 2004, open squares), where the presence of an ATP effect on bilirubin formation results in higher fidelity with the published literature for early bilirubin elevation. **(B)** Drug simulation with the ATP effect on bilirubin formation maintains consistency with the available data describing the relationship between viable hepatocytes and bilirubin (Portmann et al., 1975), circles; simulation results, line).

Acetaminophen metabolism via the cytochrome P450 system (Cyp450) yields the toxic reactive metabolite, *N*-acetyl-*p*-benzoquinone imine (NAPQI). As GSH concentration is depleted, NAPQI forms protein adducts and induces mitochondrial oxidant stress, leading to cell death. The DILIsym™ model includes the basic biochemistry to describe these processes, and model simulations are consistent with molecular data, e.g., changes in liver GSH following APAP administration (Figure 7), and the corresponding circulating indicators of liver injury, e.g., ALT (Figure 8). Formalizing the available literature in the DILIsym™ model has itself provided insights into the underlying biology. For example, the modeling team sought to examine Hy’s Law which specifies liver injury concerns in subjects with simultaneous elevations of ALT exceeding three times the upper limit of normal (ULN) and of bilirubin exceeding twice the ULN. Bilirubin is inversely correlated with viable hepatocyte numbers (Portmann et al., 1975). However bilirubin is elevated before liver necrosis is apparent (Zieve et al., 1985; Sawant et al., 2004; Pooranaperundevi et al., 2010a,b), suggesting that hepatocyte death may not be the primary mechanism underlying early increases in bilirubin. Alternate mechanisms for drug-induced loss of hepatocellular function were investigated. While analyzing these data, we observed an inverse correlation between GSH and bilirubin following drug administration (Sawant et al., 2004; Pooranaperundevi et al., 2010a,b). In addition, there is a direct correlation between hepatic GSH and ATP levels (Jenner and Timbrell, 1994). Together, these data indicate that drug-induced bilirubin elevation might initially result from a decrease in hepatocellular ATP. Bilirubin processing includes several steps that are likely ATP-dependent, e.g., bilirubin conjugation and export from the hepatocyte (Tiribelli and Ostrow, 1996; Paulusma et al., 1997; Borst et al., 2007). Using the DILIsym™ model, APAP was simulated in the presence or absence of an ATP contribution to bilirubin generation. The addition of an ATP effect more faithfully reproduces experimental data on drug-induced early bilirubin elevation than drug-induced hepatocyte death alone (Figure 9A), and inclusion of an ATP effect does not compromise consistency with the data relating hepatocyte numbers to bilirubin (Figure 9B). This example illustrates the integration of multiple datasets and the manner in which it supports the formulation of new hypotheses that better reconcile the data.

**Figure 10:**
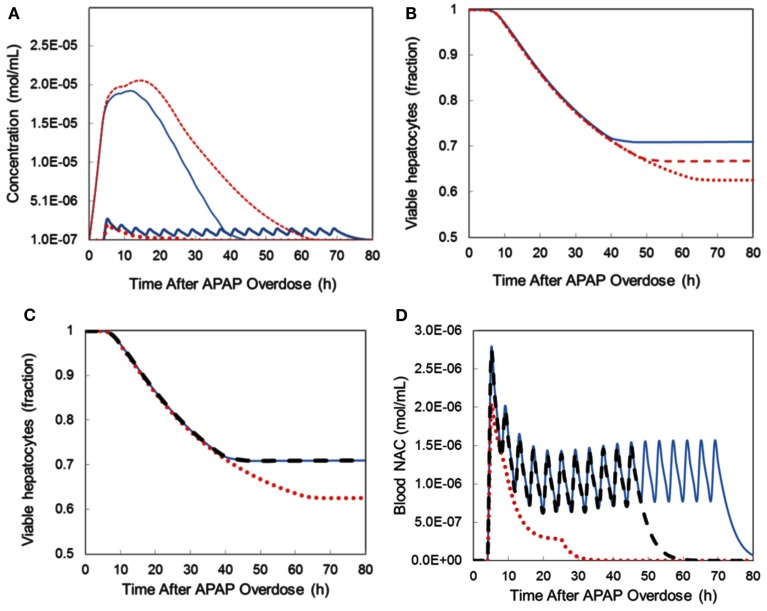
**The DILIsym™ model was applied to evaluate NAC treatment protocols following 60 g APAP overdose**. **(A)** NAC was initiated 4 h after overdose. The level of circulating NAC was compared between standard oral (thick line) and IV (dotted line) NAC protocols and compared against levels of liver NAPQI for oral (thin line) and IV (dashed line) protocols. **(B)** Hepatocyte viability was compared following application of the standard oral (thick line) and IV (dotted line) NAC protocols and with extension of the IV NAC protocol to 72 h (dashed line). **(C)** Hepatocyte viability was compared following application of the standard oral (thick line) and IV (dotted line) NAC protocols or the Smilkstein IV protocol (Smilkstein et al., 1991; dashed line). **(D)** Higher NAC levels with the Smilkstein IV protocol (dashed line) better control peak NAPQI levels than standard oral (thick line) or IV (dotted line) protocols.

The DILIsym™ model also allows protocol optimization. *N*-acetyl-cysteine (NAC) is the standard therapy for APAP overdose (Rumack et al., 1981; Heard, 2008), but there are differences in the route of administration as well as the duration of treatment (21 h intravenous vs. 72 h oral) with corresponding debate on the best treatment regime. For example, there are indications that protocol efficacy varies by the length of delay between overdose and treatment initiation (Yarema et al., 2009) and that NAC administration impedes recovery (Athuraliya and Jones, 2009; Yang et al., 2009), providing impetus to identify the shortest effective treatment. Investigative simulations were conducted comparing the standard NAC treatment protocols for 60 g APAP overdose and varying length of delay between overdose and treatment (4–44 h). The standard 72 h oral NAC protocol consistently out-performed the 21 h intravenous (IV) protocol when the delay between overdose and treatment was short; i.e., most pronounced difference in hepatocyte preservation was observed with a 4 h delay, diminishing to equivalent efficacy with longer delays (Table 1). Mechanistically, the predicted superior efficacy of the oral protocol with short delays can be attributed to the later stage of the treatment cycle, when higher levels of NAC present with the oral protocol more effectively neutralize the remaining APAP and NAPQI (Figure 10A). Prolonging the standard IV protocol such that NAC infusion continued beyond 21 h improved efficacy (Figure 10B) but did not achieve equivalence with the standard oral protocol in preservation of hepatocytes, due to the overall lower level of NAC administration used in the IV protocol.

**Table 1:** **The DILIsym™ model was applied to compare the efficacy of standard 72 h oral and 21 h IV NAC therapy following a 60 g APAP overdose and varying delays (4–44 h) between overdose and treatment initiation in the baseline human patient**.

Time elapsed between overdose and treatment (h)	72 h oral NAC (fraction of viable hepatocytes)	21 h IV NAC (fraction of viable hepatocytes)
4	0.702	0.626
9	0.615	0.546
14	0.545	0.484
19	0.487	0.440
24	0.437	0.413
29	0.387	0.372
34	0.315	0.307
39	NA	NA
44	NA	NA

*The lowest fraction of viable hepatocytes observed in the experiment was compared. NA indicates APAP overdose resulted in death of the simulated patient despite late NAC treatment (adapted from Woodhead et al., 2012)*.

Finally, we sought to identify an IV protocol that could provide equivalent efficacy to the standard oral protocol. In 1991, a group of investigators proposed a novel IV protocol which mimics the level of dosing used in the oral protocol but is condensed to 48 h duration (Smilkstein et al., 1991). They demonstrated its clinical efficacy but were unable to simultaneously evaluate it against standard protocols. Using the DILIsym™ model, side-by-side simulations confirmed that this protocol has equivalent efficacy to the standard 72 h protocol (Figure 10C). Further, simulations demonstrate that the higher NAC levels better control peak NAPQI levels accounting for the improved hepatocyte preservation (Figure 10D). This example illustrates how the DILIsym™ model may be used to compare clinical protocols under multiple scenarios (i.e., length of delay, treatment duration), understand the molecular basis of the predicted efficacy, and identify protocols that improve clinical results. Simulation results could be used to help design confirmatory clinical studies.

**Figure 11:**
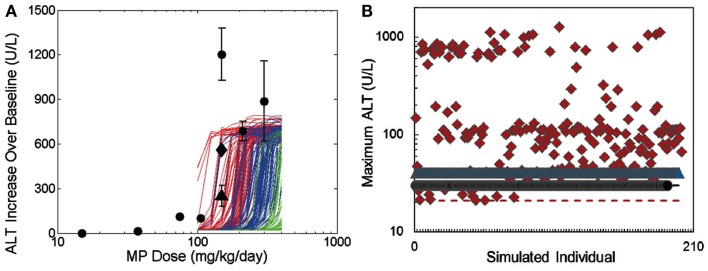
**The DILIsym™ model was evaluated for its ability to predict *in vivo* liver toxicity given only *in vitro* input data for the drug methapyrilene**. **(A)** Simulations predict a hepatotoxic threshold of 150–200 mg/kg in rats, consistent with published data (Graichen et al., 1985), circles (Ratra et al., 1998), diamonds (Ratra et al., 2000), triangles. **(B)** Biological variability represented in alternate simulated individuals yields considerable variability in the strength of liver enzyme signal in rats (diamonds), but persistently, no hepatotoxic signal in humans (circles) or mice (triangles).

The DILIsym™ model was designed to support decision making throughout the drug lifecycle, including IVIVE, in which *in vivo* outcomes are predicted using *in vitro* data. As proof-of-concept, MP was selected for evaluation. Similar to APAP, MP hepatotoxicity is thought to be mediated by a reactive metabolite, but importantly, MP differs from APAP in the observed necrotic pattern (i.e., periportal rather than centrilobular) and in species-specificity (i.e., MP toxicity in rats but not in humans vs. APAP toxicity in both rodents and humans). The model for MP was constructed using *in vitro* data, including the log P, pKa, metabolic partitioning in rat and mouse hepatocytes, and covalent binding in mouse, rat, and human microsomes. Multiple doses of MP were evaluated in simulated mice, rats, and humans. The DILIsym™ model predicted hepatotoxicity in rats between 150 and 200 mg/kg, consistent with the available literature (Figure 11A). MP hepatotoxicity has not been reported in mice; however, it was marketed in the 1950s through the 1970s with no reports of hepatotoxicity, suggesting a safe community experience. With inclusion of variability in the underlying biology, all simulated mice and humans were predicted to be tolerant to the drug (Figure 11B), while simulated rats displayed a wide range of response. This example illustrates the IVIVE capability of the DILIsym™ model.

The DILIsym™ model is a multi-species, multi-scale mechanistic model for reactive metabolite mediated DILI. The model is based upon APAP datasets, and its capabilities are illustrated above by its application to both APAP research questions (e.g., optimal NAC treatment) and related drugs (e.g., MP). Further model development is ongoing and focuses on expanding model capabilities to address other mechanisms of hepatotoxicity.

## Conclusion

We have presented some concepts relevant to implementation of a new vision for toxicity testing in the twenty-first century for chemical and pharmaceutical molecules (NAS/NRC, 2007), centered around the idea of critical perturbation to intracellular toxicity pathways and computational systems biology models to understand the topology and dynamic behavior of these pathways. Three case studies were discussed highlighting our ongoing work toward realization of the goals laid out in this vision: (i) causal network mapping of the PPARα NR pathway in primary human hepatocytes; (ii) a multi-scale agent-based model of the human liver lobule to investigate activation of the AhR pathway in liver parenchymal cells; and (iii) a predictive multi-scale physiological model (DILIsym™) to understand DILI arising from administration of acetaminophen and other drugs. These various approaches will be critical in devising *in vitro* toxicology testing strategies and determination of pathway targets, as well as improved estimation of dose-response characteristics from a network biology perspective.

## Conflict of Interest Statement

The authors declare that the research was conducted in the absence of any commercial or financial relationships that could be construed as a potential conflict of interest.
